# Asymptomatic tracheal MALT lymphoma discovered on spirometric findings presenting with elevated respiratory resistance

**DOI:** 10.1186/s13104-015-1218-5

**Published:** 2015-06-06

**Authors:** Naoki Kadota, Tsutomu Shinohara, Hisanori Machida, Hirofumi Nakanishi, Fumie Suehiro, Hiroko Toda, Tadashi Yoshino, Fumitaka Ogushi

**Affiliations:** Division of Pulmonary Medicine, National Hospital Organization National Kochi Hospital, 1-2-25 Asakuranishimachi, Kochi, 780-8077 Japan; Department of Clinical Investigation, National Hospital Organization National Kochi Hospital, 1-2-25 Asakuranishimachi, Kochi, 780-8077 Japan; Kochi Kenshin Clinic, 2-4-36 Chiyori-cho, Kochi, 780-0806 Japan; Department of Pathology, Okayama University, 2-5-1 Shikata-cho, Okayama, 700-8525 Japan

**Keywords:** Central airway obstruction, Flow–volume curve, Impulse oscillometry

## Abstract

**Background:**

Central airway obstruction (CAO) may be caused by various etiologies. However, conventional chest X-rays are rarely diagnostic for patients with CAO.

**Case presentation:**

We here described a 64-year-old asymptomatic female with tracheal mucosa-associated lymphoid tissue lymphoma discovered on spirometric findings during a complete physical examination. The plateau of forced expiratory flow was consistent with CAO. A decreased peak expiratory flow rate was noted at least 3 years before the diagnosis, and was attributed to an insufficient effort by the patient. Impulse oscillometric measurements, which were taken during quiet breathing and were effort-independent, suggested elevated respiratory resistance. These abnormalities completely disappeared after radiation therapy.

**Conclusion:**

The addition of impulse oscillometry to spirometry may be useful for screening CAO in routine health examinations.

## Background

Central airway obstruction (CAO) in the trachea and/or main bronchi may be caused by various etiologies, including a primary tumor, the formation of granulation tissue, inflammatory diseases, and foreign body aspiration. Of these disorders, tracheal malignant lymphoma is extremely rare [1–3].

The clinical presentation and disease course of CAO differs according to the underlying etiology, location, and progression rate. In any case, conventional chest X-rays are rarely diagnostic for patients with CAO.

We here described a case of asymptomatic tracheal mucosa-associated lymphoid tissue (MALT) lymphoma discovered on spirometric findings during a complete physical examination that presented with elevated respiratory resistance, as analyzed by impulse oscillometry.

## Case presentation

**Figure 1:**
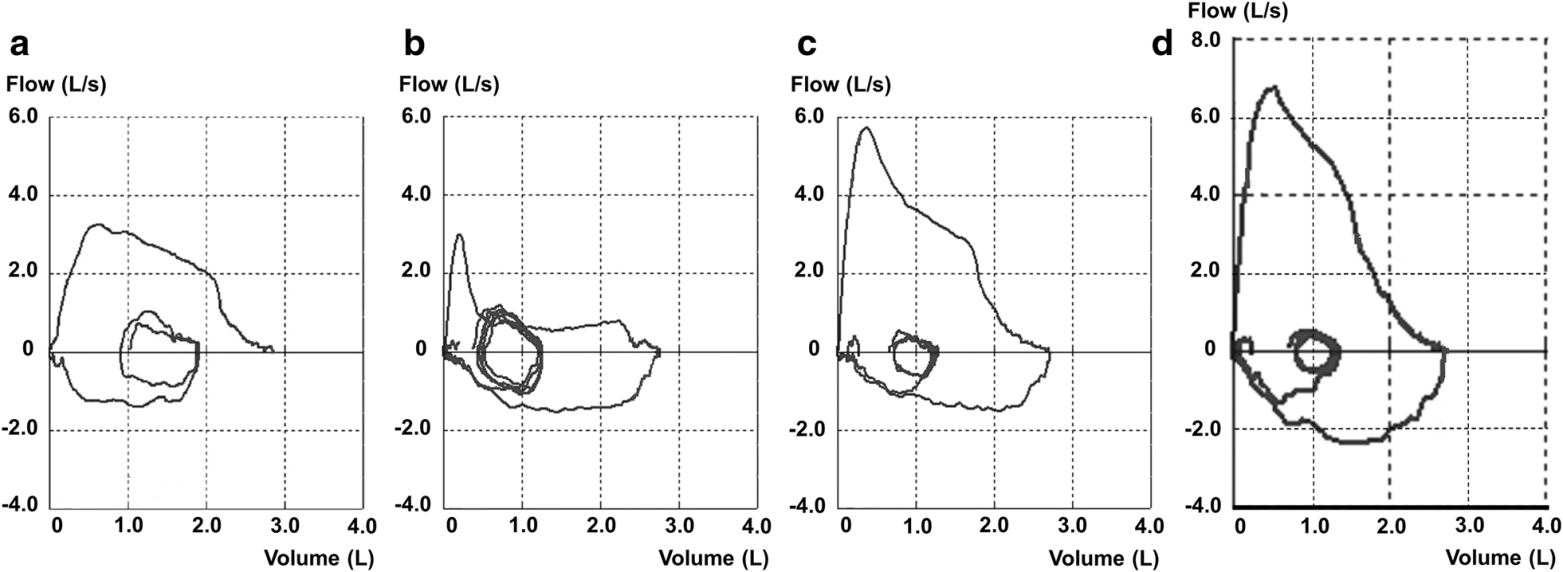
Time-course of the flow–volume curve suggesting long-term intrathoracic CAO. **a** Decreased peak expiratory flow rate 3 years before the diagnosis. **b** A plateau in FEF accompanied by relatively preserved FIF at presentation. **c** Improvements observed during radiation therapy at doses of 24 Gy. **d** Improvements observed after radiation therapy (36 Gy).

A 64-year-old female was referred to our hospital because of obstructive ventilatory impairment on spirometry (FEV_1_/FVC ratio = 36.95%, %VC = 109.3%), which was incidentally found during a complete physical examination. The pattern of a plateau in forced expiratory flow (FEF) suggested CAO (Figure 1b) [4].

**Figure 2:**
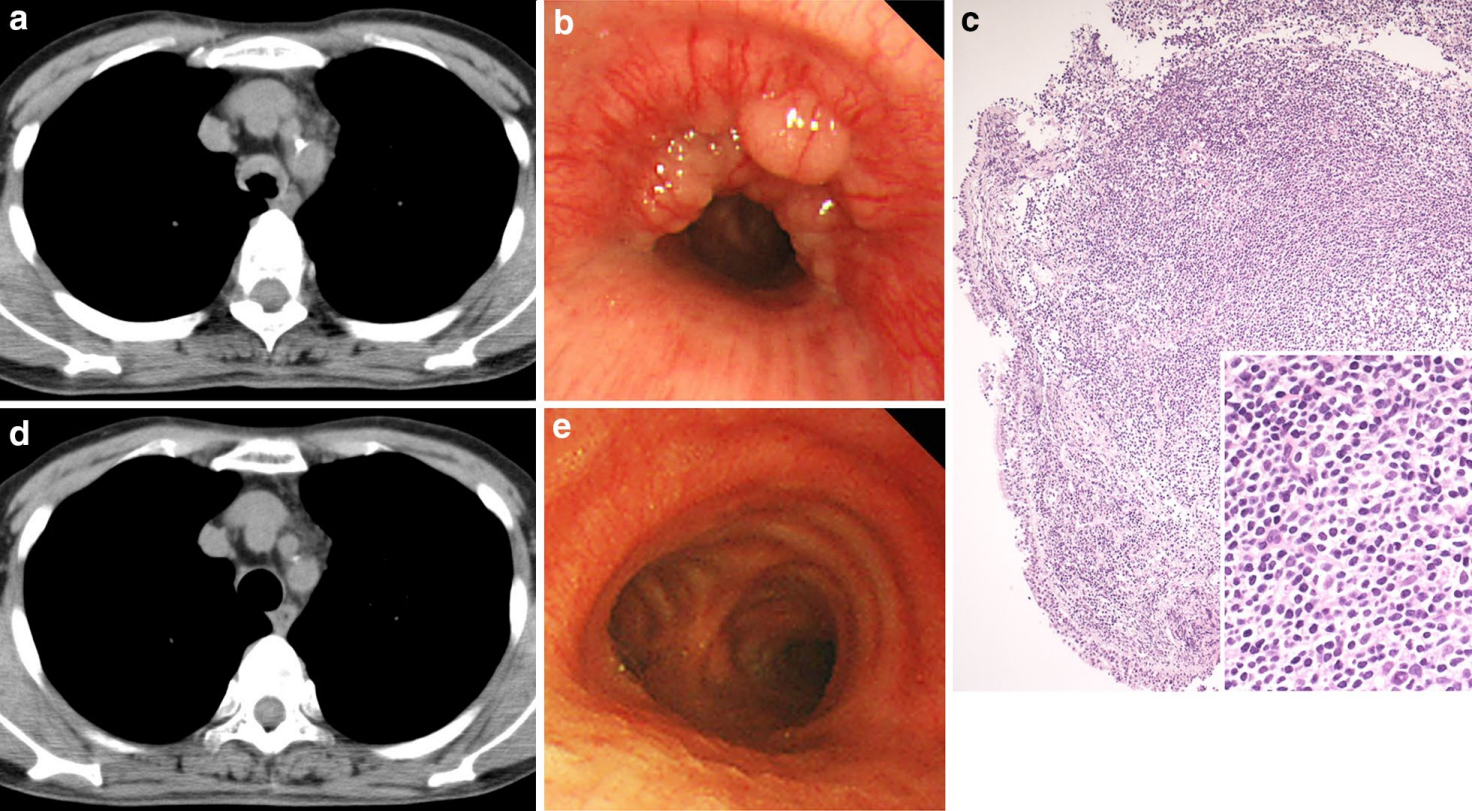
CT (**a**, **d**), endoscopy (**b**, **e**), and pathology (**c**) images before (**a**–**c**) and after (**d**, **e**) radiation therapy. **c** H&E stain ×40; *insert* ×400.

No abnormalities were observed on a chest X-ray, whereas chest computed tomography revealed anterior tracheal wall thickening (Figure 2a). The minor-axis of the airway was narrowed to approximately 9 mm, whereas the major-axis was 14 mm. An endoscopic examination revealed glossy polypoid tumors in the lower trachea (Figure 2b). The pathological findings of the tracheal mass indicated the diffuse infiltration of small- and medium-sized lymphocytes in the submucosal lesions with the formation of lymphoid follicles (Figure 2c). The malignant cells were positive for CD20, but not for CD3 or CD5, which indicated a B cell origin. The MIB-1 index in the germinal center was high. These findings established the diagnosis of endobronchial MALT lymphoma.

**Figure 3:**
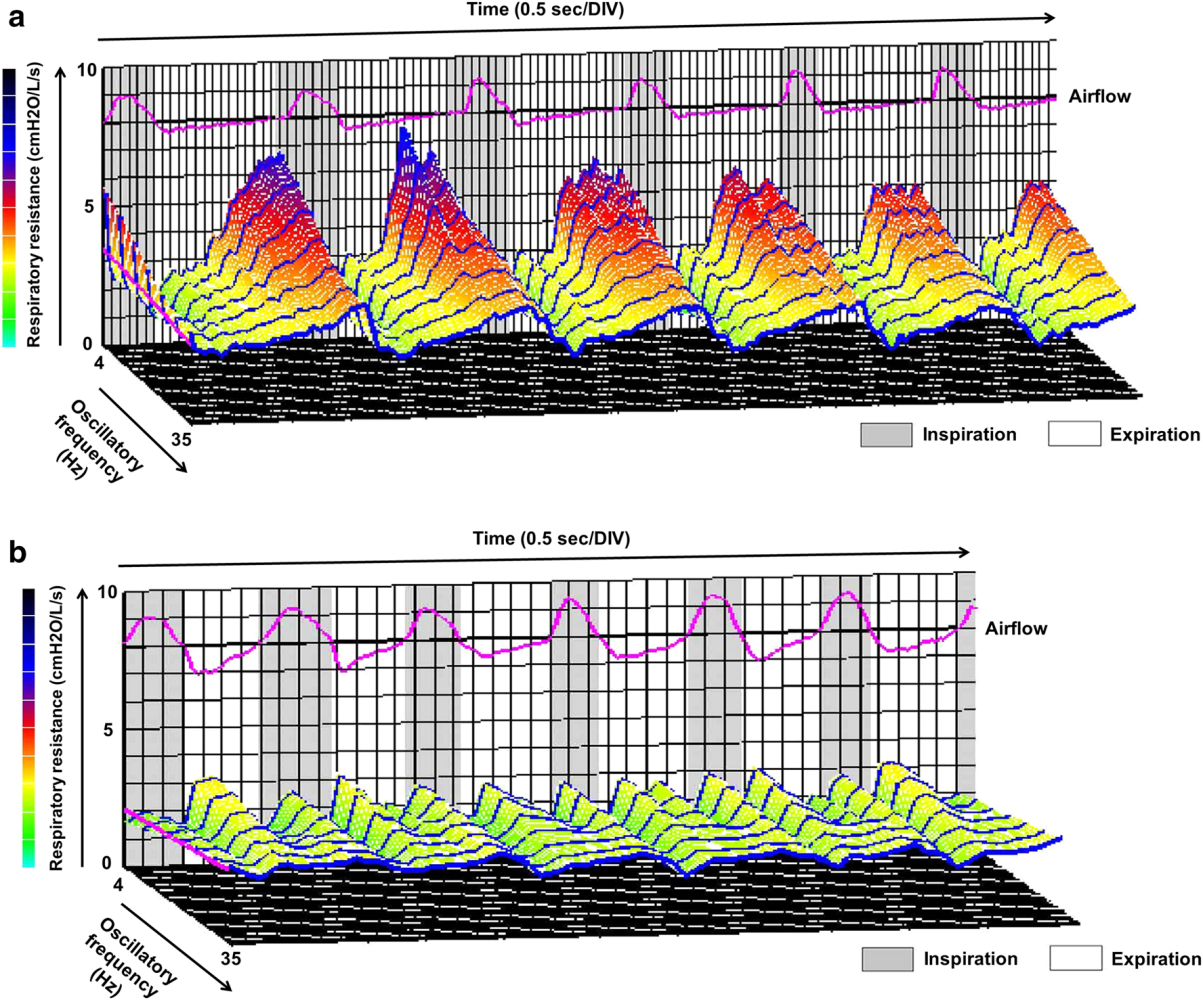
3D images of Rrs analyzed by impulse oscillometry before (**a**) and after (**b**) radiation therapy.

Retrospectively evaluated spirometric findings indicated that a decreased peak expiratory flow rate (blunting in the flow–volume curve) with a normal FEV_1_/FVC ratio (82.04%) and %VC (110%) had been recorded at least 3 years before the diagnosis (Figure 1a), and was attributed to an insufficient effort by the patient. Measurements taken by impulse oscillometry (MostGraph-01; Chest M.I., Co. Ltd., Tokyo, Japan) [5, 6] suggested elevated respiratory resistance (Rrs) (Figure 3a). The average difference between Rrs at 5 Hz (R5) and 20 Hz (R20) (0.24 cmH_2_O/L/s) and respiratory reactance (Xrs) at 5 Hz (X5) (−0.26 cmH_2_O/L/s) were small in this case.

Narrowing of the trachea completely disappeared (Figure 2d, e) and obstructive ventilatory impairment normalized (FEV_1_/FVC ratio = 85.66%, %VC = 109.8%) after radiation therapy at a total dose of 36 Gy. Abnormal findings on the flow–volume curve resolved (Figure 1c, d) and Rrs also decreased (Figure 3b).

## Discussion

The rareness of tracheal lymphoma may be ascribed to markedly less lymphoid tissue in the trachea than in the lung. MALT lymphoma commonly occurs after long-term preexisting disorders, such as gastric *Helicobacter pylori* infection, salivary gland inflammation in Sjögren syndrome, and Hashimoto’s thyroiditis. However, antigenic stimulations that induce MALT lymphoma in the trachea have not been identified. MALT lymphoma tends to localize for a long time and responds to local treatments including radiation therapy, which was selected in this case [7–11].

Exertional dyspnea and wheezing are the main symptoms of CAO. However, we need to be aware that clinical symptoms do not appear unless the trachea is narrowed to less than 8 mm [12–15]. Therefore, examining the flow–volume curve pattern in detail is crucial for detecting CAO in asymptomatic patients. The particular flattening in the flow–volume curve that implies CAO may exist previous to obstructive ventilatory impairments, as was found in this case.

Airway resistance (Raw) can be measured during body plethysmography, which is a well-established method for determining lung function using a large chamber (approximately 700–1,000 L). On the other hand, impulse oscillometry can easily evaluate Rrs and Xrs at various oscillometric frequencies without a chamber, which cannot be achieved with body plethysmography. Although the frequency of the clinical use of impulse oscillometry appears to differ between countries, this test is covered by the national health insurance of Japan. Elevated Rrs due to tracheal stenosis, as analyzed by impulse oscillometry, was previously reported in patients who had a history of tracheostomies without current tracheostomy [16, 17]. Handa et al. recently assessed CAO caused by various etiologies using impulse oscillometry and demonstrated that the R5–R20 was markedly higher in subjects with variable CAO (defined as a difference in the airway lumen diameter between inspiration and expiration >50%) than in those with fixed CAO (difference <50%), with a threshold above 2.14 cmH_2_O/L/s (0.21 kPa/L/s). Furthermore, a significant difference was observed in X5 between variable and fixed CAO, with a threshold below −1.94 cmH_2_O/L/s (−0.19 kPa/L/s) [5]. The main reason for these findings was attributed to an upper airway shunt. The R5–R20 (0.24 cmH_2_O/L/s) and X5 (−0.26 cmH_2_O/L/s) in this case suggested fixed CAO, which is consistent with malignant disease [18].

Spirometry requires a maximal effort by the patient to achieve optimal results. Inadequately performed spirometry can lead to a decreased peak expiratory flow rate and flow–volume curve similar to that in CAO, and it is difficult for non-specialist physicians to evaluate whether the spirometric test was performed based on the maximum effort of a patient. Therefore, a decreased peak expiratory flow rate with a normal FEV_1_/FVC ratio and %VC in this case was attributed to an insufficient effort by the patient. In contrast, impulse oscillometry is performed during quiet breathing and is effort-independent [19, 20]. This technique may represent a promising alternative for evaluating lung mechanics in patients in whom the maneuvers involved in spirometry and body plethysmography are difficult to carry out [16, 17]. MostGraph is a newly-developed commercially available impulse oscillometric method with color 3D imaging. Since the real-time 3D display is understandable, as shown in this case, this technology may become useful as an auxiliary tool in the diagnosis of airway diseases, even for non-specialist physicians engaged in health check-ups. However, the clinical implications of measurements taken by impulse oscillometry are still being debated. Further studies are needed in order to establish standardized guidelines for and the reliability of impulse oscillometry.

## Conclusion

The addition of impulse oscillometry to spirometry may be useful for screening CAO in routine health examinations. Moreover, malignant tracheal tumors should be considered when an abnormal flow–volume curve is detected by spirometry, even in asymptomatic subjects.

## Consent

Written informed consent was obtained from the patient for publication of this case report and any accompanying images. A copy of the written consent is available for review by the Editor-in-Chief of this journal.

## Abbreviations

CAO: central airway obstruction
MALT: mucosa-associated lymphoid tissue
FEF: forced expiratory flow
FIF: forced inspiratory flow
Rrs: respiratory resistance
Xrs: respiratory reactance
Raw: airway resistance
